# A Robust Computer-Aided Automated Brain Tumor Diagnosis Approach Using PSO-ReliefF Optimized Gaussian and Non-Linear Feature Space

**DOI:** 10.3390/life12122036

**Published:** 2022-12-06

**Authors:** Muhammad Umair Ali, Karam Dad Kallu, Haris Masood, Shaik Javeed Hussain, Safee Ullah, Jong Hyuk Byun, Amad Zafar, Kawang Su Kim

**Affiliations:** 1 Department of Unmanned Vehicle Engineering, Sejong University, Seoul 05006, Republic of Korea; 2 Department of Robotics & Artificial Intelligence (R&AI), School of Mechanical and Manufacturing Engineering (SMME), National University of Sciences and Technology (NUST) H−12, Islamabad 44000, Pakistan; 3 Electrical Engineering Department, Wah Engineering College, University of Wah, Wah Cantt 47040, Pakistan; 4 Department of Electrical and Electronics, Global College of Engineering and Technology, Muscat 112, Oman; 5 Department of Electrical Engineering HITEC University, Taxila 47080, Pakistan; 6 Department of Mathematics, College of Natural Sciences, Pusan National University, Busan 46241, Republic of Korea; 7 Department of Intelligent Mechatronics Engineering, Sejong University, Seoul 05006, Republic of Korea; 8 Department of Scientific computing, Pukyong National University, Busan 48513, Republic of Korea; 9 Interdisciplinary Biology Laboratory (iBLab), Division of Biological Science, Graduate School of Science, Nagoya University, Nagoya 464-8602, Japan

**Keywords:** ReliefF, optimization, tumor, KAZE, diagnosis, brain MRI

## Abstract

Brain tumors are among the deadliest diseases in the modern world. This study proposes an optimized machine-learning approach for the detection and identification of the type of brain tumor (glioma, meningioma, or pituitary tumor) in brain images recorded using magnetic resonance imaging (MRI). The Gaussian features of the image are extracted using speed-up robust features (SURF), whereas its non-linear features are obtained using KAZE, owing to their high performance against rotation, scaling, and noise problems. To retrieve local-level information, all brain MRI images are segmented into an 8 × 8 pixel grid. To enhance the accuracy and reduce the computational time, the variance-based k-means clustering and PSO-ReliefF algorithms are employed to eliminate the redundant features of the brain MRI images. Finally, the performance of the proposed hybrid optimized feature vector is evaluated using various machine learning classifiers. An accuracy of 96.30% is obtained with 169 features using a support vector machine (SVM). Furthermore, the computational time is also reduced to 1 min compared to the non-optimized features used for training of the SVM. The findings are also compared with previous research, demonstrating that the suggested approach might assist physicians and doctors in the timely detection of brain tumors.

## 1. Introduction

The roles of artificial intelligence, machine learning, and image processing, especially in medical diagnostics, are considered fundamental by many researchers worldwide [1]. Early and accurate diagnosis of diseases, such as brain tumors, is critical, especially considering their life-threatening nature. Machine learning and artificial intelligence algorithms are known not only for their classification abilities, but also for their data regression ability; such traits make them ideal candidates for use in brain tumor classification.

Brain tumors are considered the most life-threatening tumors by medical professionals, as they lie inside the most delicate part of the body, that is, the human brain. Once the tumor starts to manifest in the brain, it can cause fatalities for the patient. Therefore, the early detection of brain tumors is fundamental and critical. Recent studies have shown that the size of brain tumors doubles after just three and a half weeks [2]. Researchers and medical scientists believe that if computer-aided intelligent solutions are not explored, brain tumors can be fatal and life-threatening. To address this issue, there is a need for machines that can mimic human brain activity so that intelligent solutions can be provided and tested on such machines.

Brain tumors have a built-in ability to not only affect the localized area of their existence, but also start to affect the surrounding areas as well as the passage of time. Before any surgical procedure is performed, the segmentation of healthier tissues from the affected tissues is one of the trickiest, yet most important procedures. Failure to isolate affected tissues from healthier tissues can cause severe consequences, potentially including death.

Thus far, many algorithms have been developed for brain segmentation, which can be categorized as automatic, semi-automatic, and manual. To diagnose tumors effectively, information regarding their size, shape, and location is required [3]. Brain tumors are classified as benign or malignant based on their location, progression stage, type, and pace of growth [4,5]. In benign brain tumors, the affected cells seldom assault healthy cells. They also develop slowly and have distinct boundaries, similar to meningiomas and pituitary tumors. In contrast, in malignant brain tumors, damaged cells affect the nearby healthy cells. These tumors, like gliomas, can progress rapidly and have a wide range of restrictions. As a result, early detection of cancer types (meningioma, pituitary, and glioma) is critical for medical care to save patient lives.

The most common medical imaging techniques currently used are single-photon emission computed tomography (SPECT), magnetic resonance imaging (MRI), magnetic resonance spectroscopy (MRS), and computed tomography (CT) [6]. MRI, a non-invasive technique, is considered the most effective technique for medical imaging [7]. MRI provides a large number of diverse high-contrast 2D images that can be used for brain tumor segmentation. The high soft-tissue contrast provided by MRI makes it an ideal technique for the detection of abnormal brain tissues. Advanced studies have presented the use of different MRI modalities, each providing images with varying tissue contrast, thus providing more flexibility in image analysis. In addition to presenting highly contrasting diverse images, MRI also demonstrates the ability to accurately determine the location of the tumor, a trait that many of its counterparts do not possess. Lesions in fundamental neuroanatomic structures can also be successfully identified using MRI. However, manual MRI scan interpretation is time-consuming and rife with errors. An automatic computer-aided diagnostic (CAD) approach is needed to detect brain damage.

The development of machine learning techniques has improved the effectiveness of CAD systems in assisting physicians in diagnosing brain cancers [8,9,10,11]. To identify brain tumors, a variety of learning techniques have been proposed in the literature, which can be further divided into deep and conventional learning techniques [12]. Convolutional neural networks (CNNs) are typically used in deep learning techniques for the detection of brain tumors using MRI [13]. Numerous researchers have employed developed and pre-trained deep learning models to classify cerebral MRI images. One study [14] created a CNN classifier model that classifies MRI images obtained from the brain into two categories (i.e., tumor vs. no tumor). The detection of tumor subtypes was the biggest failure of the model; its fundamental limitation was that it could not classify brain tumors into subgroups. A CNN model was created to identify different classes of brain tumors (glioma, meningioma, and pituitary) [15]. However, the model accuracy was only 84.19%. Recently, Badza and Barjaktarovic [6] classified brain MRI images into three categories using a CNN classifier model. To improve the categorization accuracy, the investigators also applied data augmentation. A 10-fold cross-validation strategy resulted in a classification accuracy of 96.56%. However, the literature shows that although data augmentation helps enhance the classification accuracy, its reliability for real-time applications has not yet been proven. In another study [16], the authors created a 25-layer CNN model with a 92.66% accuracy rate to categorize brain MRI images into five categories. To categorize brain MRI images, pre-trained available networks (i.e., GoogLeNet and ResNet-50) were also employed [17,18,19]. However, deep networks require a lengthy training period, a complicated design, large memory demands, and powerful graphics processing units (GPU), among other drawbacks.

Conventional models, in contrast to deep learning models, require the most fundamental aspects of brain MRI scans to identify brain tumors. As a result, they require less model training time; examples include support vector machines (SVMs), tree, and Naïve Bayes. In a previous study [20], the author calculated the gray-level co-occurrence matrix of brain MRI images and divided them into two groups. The accuracy of the model was high; however, the investigator could only find a tumor in the brain MRI images and could not identify tumor subclasses. Because brain MRI images are similar, the accuracy of these global-level features for tumor subtype identification is not very high. Additionally, global-level features such as texture that are extracted through a gray-level co-occurrence matrix, histograms of oriented gradients, and local binary patterns, among others, are quite sensitive to noise, scaling, rotation, and visibility, all of which have an impact on performance, memory usage, and execution time, among other metrics. Scale-invariant feature transformation, Fisher vectors, and the bag-of-words model [21] are examples of local-level features which can aid in identifying brain MRI images [21,22,23]. In a previous study [24], the authors used the histogram intensity, the bag-of-words model, and the gray-level co-occurrence matrix to identify MRI brain images. For the three-class classification brain MRI dataset, a 91.28% classification accuracy was obtained. In a recent study [25], the authors used pre-trained CNN models to compute the deep features of brain MRI image datasets. The findings demonstrated that the hybrid features of the pretrained model, when used with an SVM classifier, had the highest accuracy (93.72%). However, because of the length of the feature vector, training took a long time. In a study by Almalki et al. [26], speed-up robust features (SURF) and KAZE features were combined to create a hybrid training feature vector that was used to categorize brain MRI images. The findings demonstrated that the proposed model has a high computational cost and an accuracy of 95.33%. Consequently, considering the drawbacks of deep learning and conventional learning techniques in terms of architectural complexity, memory and data processing requirements, lengthy computation time, scalability, rotation, noise, and visibility, further research is needed to detect and differentiate brain tumors.

In this study, the SVM model is trained using features extracted from brain MRI images within the Gaussian scale space using speed-up robust features (SURF) and non-linear scale-space using KAZE. A grid size of 8 × 8 pixels is used to retrieve local-level information from brain MRI images. To improve the classification performance and decrease the computational time and memory requirements, redundant Gaussian (SURF) and non-linear (KAZE) features are removed using the k-clustering and PSO-ReliefF algorithms. Finally, the proposed technique is validated using an internet-accessible dataset. To validate the proposed technique, conventional classifiers are trained using an online dataset that is readily available. Finally, a comparison between the findings of this study and other established models is performed.

The remainder of this paper is structured as follows. Section 2 includes details about the dataset used in this study. The feature extraction and framework of the proposed technique are explained in Section 3. Finally, Section 4, Section 5 and Section 6 present, discuss, and summarize the findings.

## 2. Brain Experimental MRI Dataset

This study uses an online database of brain MRI images to validate the proposed framework. The dataset for this study was collected from the Kaggle website [27]. It has one no-tumor class and three tumor subclasses: glioma, pituitary, and meningioma. It contains 2870 brain MRI images. Table 1 contains further information about the dataset.

## 3. Materials and Methods

### 3.1. Extraction of Features

A prominent topic in computer-aided image processing is feature extraction and description. In the case of various image variations, it is vital to calculate the repeatable and distinctive properties of the image for high-accuracy image classification applications. Brain tumor classification is likewise mostly based on the extraction of relevant and associated information from brain MRI images. As a result, various features from the local [22,23] and the global levels [20] are utilized to categorize brain MRI images. By contrast, in a multiclass framework, global-level features have accuracy difficulties, as mentioned in Section 1. Therefore, several local-level features, including KAZE [28], scale-invariant feature transform (SIFT) [29,30], and speeded-up robust feature (SURF) [31], calculate distinguishing features at diverse and relevant discrete points. These distinguishing characteristics are mostly related to the local mean/minima/maxima of the calculated features. The intensity of these points of interest can be described using a descriptor vector. The SIFT descriptor feature vector was first introduced by Lowe in 1999 [29,30]. Because of its invariance to rotation characteristics, translation invariance, resilience to noise, and scale invariance, it has attracted considerable attention. SIFT feature extraction is not recommended for real-time applications because of its high computational cost [32].

#### 3.1.1. KAZE

Nonlinear diffusion and the additive operator splitting method are both used in the ground-breaking 2D feature extraction and description technique known as KAZE [28]. Consequently, image blurring may be precisely adjusted to feature locations, which reduces noise without changing the borders of the image region. The Hessian Matrix Determinant is used to compute the KAZE at various scale levels using a normalized scale. The mean/minima/maxima of the signal intensity are identified as feature points using a moving window. By identifying the dominant orientation in a circular area around each identified feature, the rotational invariance trait can be integrated into the feature description. With a negligible increase in processing cost, it possesses the characteristics of rotation and scale invariance, low invariance to affine, and greater distinctness at various scales.

The nonlinear diffusion equation can be written as.

(1) $$\frac{\partial L}{\partial t}=div\left(c\left(m,n,t\right).\nabla L\right)$$

where

$$\begin{array}{l} c=\;\mathrm{conductivity}\;\mathrm{function}\\ div=\;\mathrm{divergence}\\ \nabla =\;\mathrm{gradient}\;\mathrm{operator}\\ L=\;\mathrm{image}\;\mathrm{luminance} \end{array}$$

#### 3.1.2. Speeded Up Robust Feature (SURF)

The SURF technique was developed by Bay et al. [31] in order to address the robustness problems of the SIFT approach. Identical to the SIFT technique, the SURF technique relies on Gaussian scale-space image processing; unlike the SIFT detector, however, the SURF technique relies on the Hessian Matrix determinant. Integrated images are used to accelerate local feature extraction. Every identified feature is described by SURF’s 64-bin descriptor, utilizing the dispersal of Haar wavelet responses in a particular region. The SURF features exhibit minimal affine invariance in contrast to SIFT; however, the descriptor can be extended to 128-bin values to handle more significant perspective alterations. At point “m=(m,n)” at scale “σ”, the Hessian Matrix can be created as,

(2) $$H(m,\sigma )=\left[\begin{array}{cc} L_{mm}(m,\sigma ) & L_{mn}(m,\sigma )\\ L_{mn}(m,\sigma ) & L_{nn}(m,\sigma ) \end{array}\right]$$

where $L_{mm}(m,\sigma )$ = Gaussian second order derivate convolution $\frac{\partial^{2}}{\partial x^{2}}g(\sigma )$ with the image *I* at a point *m*.

### 3.2. Feature Vector Dimension Reduction Using ReliefF

The quality and quantity of the features are the factors that matter the most in all machine-learning-based categorization techniques. A few irrelevant features offer only scant information, resulting in low accuracy. Under these circumstances, it is difficult for learning techniques to be correctly executed. Therefore, to improve the performance of the classification model, a small selection of important features must be extracted and used to characterize the targeted classes.

To address this issue, Kira and Rendell [33] developed a method that employs instance-based learning to choose the most pertinent feature of the entity for binary classification tasks. Kononenko [34] introduced ReliefF, an extension of the Relief method for multiclass tasks. The algorithm performs satisfactorily in a disturbed environment. Algorithm 1 shows the working framework of the ReliefF method.
**Algorithm 1** Working framework of ReliefF [35,36].***Input***: for each training instance a vector of attribute values and the class value. ***Output***: the vector *W* of estimations of the qualities of attributes.
1. set all weights *W* [A] := 0.0;
2. **for**
*i* := 1 to *n*
**do begin**
3.  randomly select an instance *R_i_*;
4.  find *k* nearest hits *H_j_*;
5.  **for** each class C ≠ class (*R_i_*) **do**
6.      from class C find *k* nearest misses *M_j_*(*C*);
7. **for** A := 1 to a **do**
8. $W[A]:=W[A]-\sum_{j=1}^{l}diff(A,R_{i},H_{j})/(n\cdot k)+\sum_{C\neq class(R_{i})}^{l}[\frac{P(C)}{1-P(class(R_{i}))}]\sum_{j=1}^{l}diff(A,R_{i},M_{j}(C))/(n\cdot k)$ 9. **end**;

First, initialize instance (*R_i_*) randomly. Next, for each class (nearest hits (*H_j_*)) and all other remaining classes (known as nearest misses *Mj*(*C*)), it will look for *k* to its nearest neighbor. Finally, it revises the equations shown in points 7, 8, and 9 in Algorithm 1; additional information on the ReliefF method can be found in [35,36]. Weight adjustment is substantially influenced by the value of *k*. Similarly, the feature quantity (the number of features for the training vector) also has a significant influence on model accuracy. Therefore, particle swarm optimization (PSO) is used in this study to determine the ideal value of *k* and the feature vector size.

### 3.3. Particle Swarm Optimization

Particle swarm optimization is a population-based optimization technique that draws inspiration from the teamwork of fish schools and bird flocks [37,38]. The PSO calculates the ideal solution by increasing or decreasing the problem. In this method, information is disseminated among groups while they look for nearby goal. Despite not knowing the precise location of the meal, they all arrived at the same spot because of information sharing.

According to the boundaries of the feature vector, the population (the feature vector size and the value of *k*) is randomly initialized in the PSO for the ReliefF-based technique. The population and its associated velocities are initialized in this study by selecting 10 combinations. The cost function is then calculated for the individual particles using Equation (3) to obtain the fitness value for the cost function.

(3) $$\min\;(F)=\frac{\mathrm{Total}\;\mathrm{no}.\;\mathrm{of}\;\mathrm{actual}\;\mathrm{images}\;-\;\mathrm{Total}\;\mathrm{no}.\;\mathrm{of}\;\mathrm{true}\;\mathrm{classfied}\;\mathrm{images}}{\mathrm{Total}\;\mathrm{no}.\;\mathrm{of}\;\mathrm{actual}\;\mathrm{images}}$$

Calculate each particle’s best location $\left(P_{best}\right)$ based on the fitness value of each particle, and then update it repeatedly. Then, compare all the values of $\left(P_{best}\right)$ for each particle, which are likewise updated repeatedly, to determine the global best position $\left(G_{best}\right)$. Based on $P_{best}$ and $G_{best}$, the velocity of each particle can be calculated as follows:

(4) $$v_{ij}^{t}=\omega v_{ij}^{t-1}+c_{1}r_{1j}^{t-1}\left[P_{best}-x_{ij}^{t-1}\right]+c_{2}r_{2j}^{t-1}\left[G_{best}-x_{ij}^{t-1}\right]$$

where

$$\begin{array}{l} v_{ij}^{}=\;\mathrm{velocity}\;\mathrm{of}\;\mathrm{each}\;\mathrm{partilce}\\ x_{ij}^{}=\;\mathrm{poisition}\;\mathrm{of}\;\mathrm{each}\;\mathrm{partilce}\\ c_{1}^{}=\;\mathrm{cognitive}\;\mathrm{parameter}\\ c_{2}^{}=\;\mathrm{social}\;\mathrm{parameter}\\ \omega =\;\mathrm{initial}\;\mathrm{weight}\\ r_{1}^{}=\;\mathrm{random}\;\mathrm{variable}\\ r_{2}^{}=\;\mathrm{random}\;\mathrm{variable} \end{array}$$

The following equation can be used to update each particle’s location after determining their separate velocities.

(5) $$x_{ij}^{t}=x_{ij}^{t-1}+v_{ij}^{t}$$

**Figure 1:**
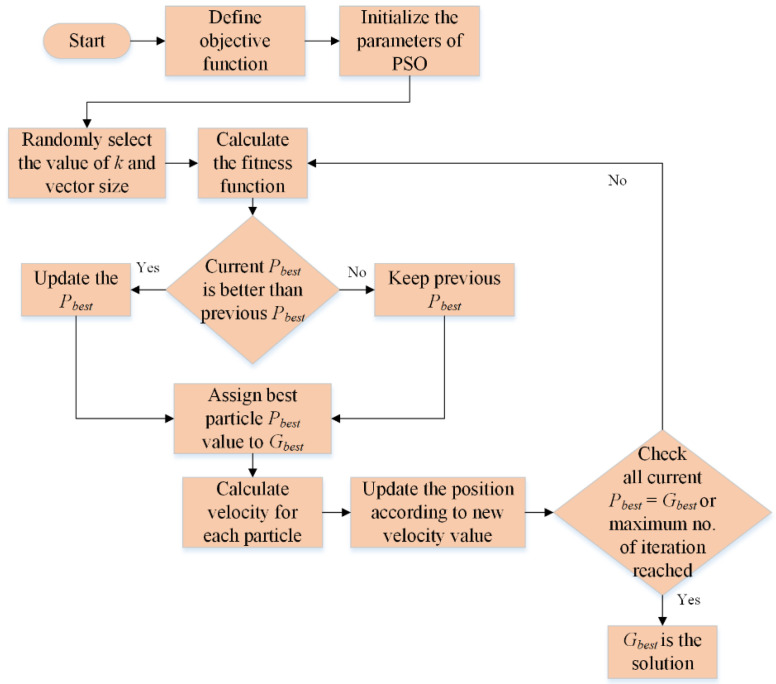
PSO flowchart for determining the ideal value of *k* and feature vector size.

The above procedure will continue until either the algorithm meets the maximum iteration requirement, or all the particles converge to a single value. Figure 1 shows the PSO’s complete flowchart. For more information about PSO, see [39,40].

### 3.4. Support Vector Machine (SVM)

The SVM model was first presented by Cortes and Vapnik [41] in 1995, and it is now a highly popular and efficient classifier utilized in many domains [42,43,44]. The non-linear input data space (i.e., low dimensional) is converted into a linear high-dimensional data space using the SVM method using kernel functions $K(x,x_{a})$. Equation (6) provides the function of the hyperplane that was used to split the transmitted data into a high-dimensional linear data space.

(6) $$y(x)=\sum_{a=1}^{n}\beta_{a}K(x,x_{a})+b_{1}$$

The data can be classified using a variety of kernel functions, including the linear, sigmoid, and RBF kernels. For more information about SVM, refer to [41,43].

### 3.5. Proposed Framework

**Figure 2:**
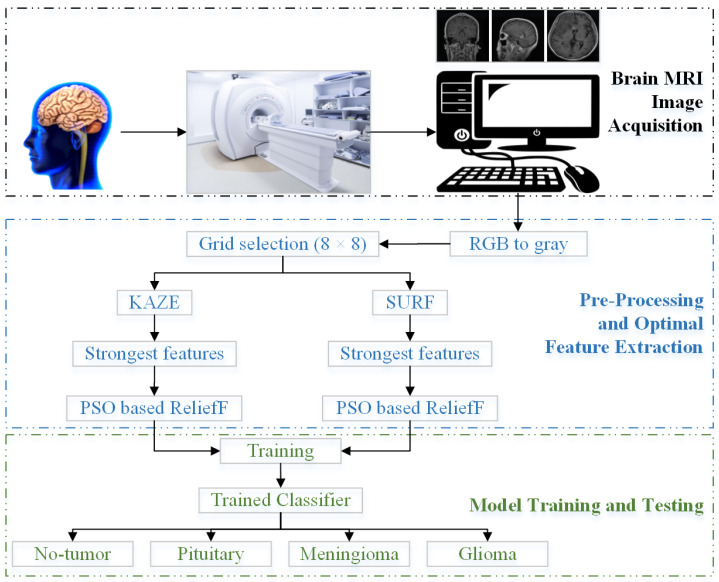
A proposed framework to categorize brain MRI images.

The architecture of the proposed methodology is discussed in detail in this section. As illustrated in Figure 2, the proposed method comprises five key parts: collection of brain MRI images, preprocessing, feature extraction, optimal feature selection, and model training.

Brain MRI equipment is used to acquire brain images in the first stage. The collected brain MRI images are then converted from RGB to grayscale using a pre-processing software. The feature extraction selection spot of the MRI images of the brain is then established as an 8 × 8 pixel grid. The computational complexity and input vector size vary with pixel size. Additionally, the KAZE and SURF features are extracted using the four-dimensional vectors (16, 32, 48, 64) and (17, 34, 51, 68), respectively, according to the literature [26]; KAZE and SURF extraction are discussed in Section 3.1. The feature vector size is then decreased by 20% by removing unnecessary features. The k-means clustering technique is used for feature segmentation owing to its simplicity and resilience. Additionally, it can maintain observations within each cluster as far apart as possible from objects in other clusters. As a result, the k-means clustering method is employed to obtain 400-feature histograms for both KAZE and SURF individually. For more information on the k-means clustering method, refer to [45,46]. Subsequently, the best features and vector size are computed using the PSO-ReliefF method, as explained in Section 3.2 and Section 3.3, to improve classification performance.

The models are then trained using several machine learning classifiers, such as SVM, tree [47], Naïve Bayes [48], k-nearest neighbors (K-NN) [49], ensemble, and neural network (NN). The findings of the proposed method are presented in the next section.

## 4. Results

**Figure 3:**
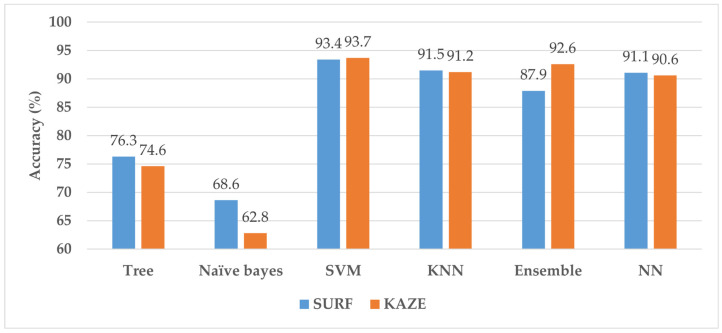
The performance of SURF and KAZE-trained machine learning models with PSO-ReliefF.

MATLAB 2021 is used in this study to train the classifier models on a computer running the 64-bit Windows 11 operating system having technical specifications of an Intel Core i7 11th generation processor, 32 GB RAM, NVIDIA GeForce GTX 1060 GPU, and 1 TB SSD storage. Only 80% of the images from each category are used for model training; the remaining 20% of the images are used to check the performance of the trained models. The classification accuracy is employed as a statistic for comparing the various trained models. Figure 3 depicts the outcomes of the KAZE- and SURF-trained models without PSO-ReliefF.

**Figure 4:**
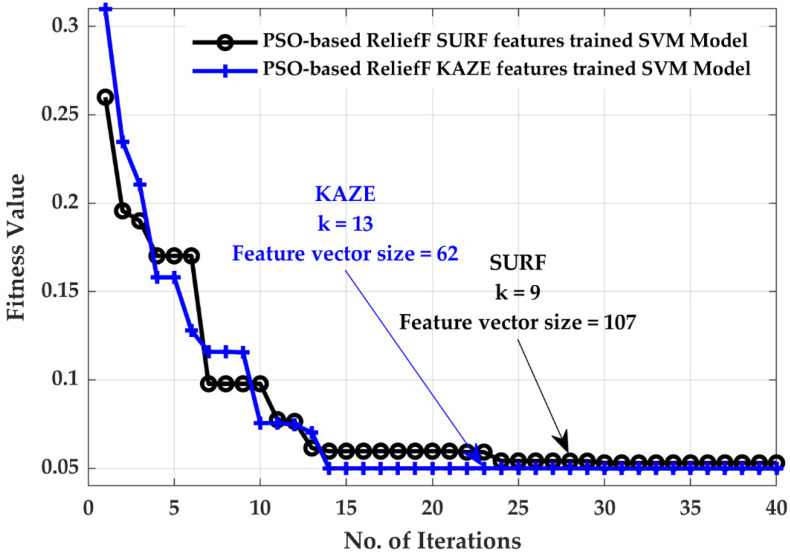
Convergence curves.

Figure 3 demonstrates that the SURF and KAZE feature-trained SVM have the highest accuracy among all models, with 93.4 and 93.7% accuracy, respectively. Further details regarding these results are provided in [26]. For the SURF- and KAZE-based SVM classifiers, the PSO-ReliefF algorithm is used to improve the model performance while reducing the feature vector size. Figure 4 illustrates the PSO-ReliefF convergence curves for both SVM models (SURF and KAZE).

After closely examining the convergence curves, it is clear that the fitness function has a high value at the beginning of the PSO algorithm. As the number of iterations of the algorithm increases, the value of the cost function began to decrease. As described in Section 3.3, as the algorithm begins to tune its parameters, all the particles begin to converge towards the global optimum value. Finally, for the SURF-based SVM model, the approach converges to a fitness value of 0.053, for *k* of 9 and a feature vector size of 107. Similarly, with *k* = 13 and a feature vector size of 62, the KAZE-based SVM model converges to a fitness value of 0.0498. The models are further evaluated using true positive rate (TPR), false negative rate (FNR), positive predictive value (PPV), and false discovery rate (FDR). The equations for computing the TPR, FNR, PPV, and FDR are provided in Equation (7).

(7) $$\begin{array}{l} \mathrm{TPR}=\frac{\mathrm{True}\;\mathrm{positive}}{\mathrm{No}.\;\mathrm{of}\;\mathrm{real}\;\mathrm{positive}}\\ \mathrm{FNR}=\frac{\mathrm{False}\;\mathrm{negative}}{\mathrm{No}.\;\mathrm{of}\;\mathrm{real}\;\mathrm{positive}}\\ \mathrm{PPV}=\frac{\mathrm{True}\;\mathrm{positive}}{\mathrm{True}\;\mathrm{positive}\;+\;\mathrm{False}\;\mathrm{positive}}\\ \mathrm{FDR}=\frac{\mathrm{False}\;\mathrm{positive}}{\mathrm{True}\;\mathrm{positive}\;+\;\mathrm{False}\;\mathrm{positive}} \end{array}\}$$

Table 2 and Table 3 show the detailed results of the PSO-ReliefF-trained SVM model for SURF and KAZE features.

After the PSO-ReliefF SURF and KAZE-trained SVM models achieve 94.70% and 95.02% accuracy, respectively, it may be considered advantageous to merge both features to construct a hybrid model to classify brain MRI images. Table 4 presents the results for the hybrid model.

**Figure 5:**
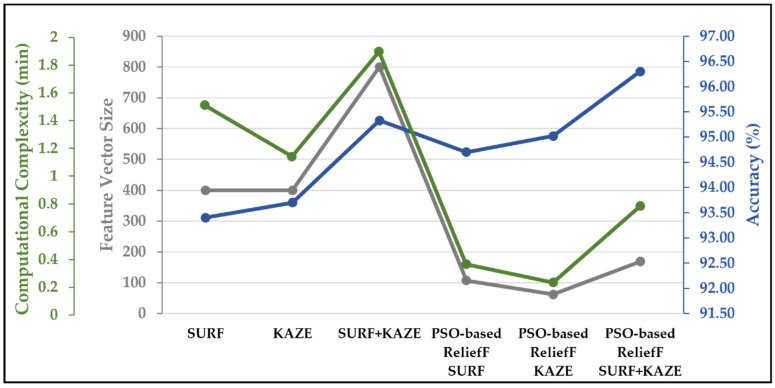
Comparison of SURF, KAZE, PSO-ReliefF SURF, PSO-ReliefF KAZE, and PSO-ReliefF SURF + KAZE (proposed approach) trained SVM models.

The accuracy of the SVM trained using concatenation features is 96.30%, which is almost 3% and 1.28% better than that of the SVM trained with only SURF and only PSO-ReliefF SURF features, respectively (see Figure 3 and Table 2 and Table 4). As a result, the presented PSO-ReliefF SURF + KAZE developed SVM model has a TPR of 95.88% for glioma and 99.40% for pituitary tumors (see Table 4). Furthermore, compared to the PSO-ReliefF KAZE developed model, the proposed approach properly identifies 18 more no-tumor class MRI brain images (see Table 3 and Table 4). Compared to the PSO-ReliefF SURF developed model, 31 more brain MRI images are properly categorized as belonging to the meningioma tumor class. The computational complexity, feature vector size, and accuracy comparison of the SURF, KAZE, PSO-ReliefF SURF, PSO-ReliefF KAZE, and PSO-ReliefF SURF + KAZE (proposed approach) feature-trained SVM models are shown in Figure 5. The SVM-trained SURF and KAZE models use 400 features each. The SURF-trained SVM model requires almost 1 min 30 s to achieve an accuracy of only 93.40%, whereas KAZE requires 1 min and 8 s to yield an accuracy of 93.7%. In comparison, the computational time is reduced to almost 22 s and 14 s for the PSO-based ReliefF SURF and PSO-based ReliefF KAZE models, respectively. The hybrid (proposed) model requires approximately 47 s with 169 features and shows the highest classification accuracy of 96.3%.

The findings of the presented scheme are also in contrast to those obtained using the cutting-edge techniques described in the literature. Table 5 compares the proposed tumor diagnostic model with the previously used methods based on their accuracy.

## 5. Discussion

A computer-based method known as CAD helps doctors make snap decisions in the area of medical imaging. Various researchers have reported training techniques to classify brain MRI images [6,16,22,25,51].

In this study, a brain tumor classification SVM model developed with PSO-ReliefF SURF + KAZE features were proposed using brain MRI images. The SURF and KAZE features were first extracted from the collected brain MRI data using an 8 × 8 pixel uniform grid, as explained in Section 3.1.1 and Section 3.1.2. The whole-brain MRI dataset of various classes was therefore retrieved, yielding 16,577,120 features. In addition, the feature vector size of the complete dataset was decreased to 7300,864 by computing 80% of the strongest features using the computer vision toolbox of MATLAB. Then, for each image, k-means clustering was used to separately generate a 400-feature vector for SURF and KAZE. Subsequently, the PSO-ReliefF algorithm was implemented to disregard the redundant SURF and KAZE features. As depicted in Figure 4, PSO-ReliefF converges to a fitness value of 0.053, with *k* = 9 and the size of the feature vector = 107 for the SURF feature; similarly, the KAZE feature has a vector size of 62, with *k* = 13 for a fitness value of 0.0498. Finally, the SVM model was trained using the optimal features of both descriptors (SURF + KAZE), which demonstrates that the proposed model has the highest accuracy of 96.30% while having an acceptable calculation time of only 0.7856 s and a vector size of only 169 values (see Figure 5). As depicted in Figure 5, the authors also perform a comparative analysis of SURF-, KAZE-, PSO-ReliefF SURF-, PSO-ReliefF KAZE-, and PSO-ReliefF SURF + KAZE (proposed approach)-trained SVM models. The proposed approach improves accuracy by 1%, reduces computation time by 1 min 1 s, and reduces feature vector size by 631, when compared to the standard SURF + KAZE-trained SVM model [26]. The developed model also shows better results than previously published works [16,19,24,25,26,50,51] (see Table 5).

The improvement in complexity and computation time enables the proposed scheme to be easily implemented on a low-cost portable embedded platform. Once the images are obtained from imaging, modality can be directly fed to the embedded platform for real-time classification of brain tumors. As a result, the presented method could be helpful in aiding clinicians and doctors in the early diagnosis of brain tumors.

## 6. Conclusions

This study used brain MRI images to provide an automated brain tumor diagnosis method. The SURF and KAZE features were first computed at an 8 × 8 pixel grid in brain MRI images. Subsequently, segmentation using k-means clustering was performed to collect 80% of the strongest features. Then, PSO-ReliefF was used to minimize the feature vector size and improve the model performance. An increase of almost 1.3% was noted in the performance of the SURF and KAZE models using PSO-ReliefF with an almost 2.5 times smaller training vector size. Furthermore, the features of both descriptors (SURF + KAZE) were merged to form a new training vector, yielding a brain MRI image classification accuracy of 96.30%. The proposed technique outperformed the findings reported in extant literature owing to its high accuracy and shorter calculation time. As a result, the proposed method can be utilized to automatically detect brain tumors.

## Figures and Tables

**Table 1:** Details about brain MRI dataset available on Kaggle website [27].

Category	Brain MRI Images	No. of Brain MRI Images
No-tumor	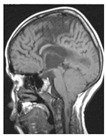	395
Glioma Tumor	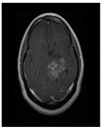	826
Meningioma Tumor	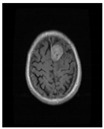	822
Pituitary Tumor	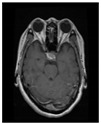	827

**Table 2:** Performance of PSO-ReliefF SURF-trained SVM model.

Class	Classified as				TPR (%)	FNR (%)	PPV (%)	FDR (%)	Accuracy (%)
	Glioma Tumor	Meningioma Tumor	No- Tumor	Pituitary Tumor					
Glioma Tumor	779	47	0	0	94.31	5.69	97.13	2.87	94.70
Meningioma Tumor	22	744	35	21	90.51	9.49	91.63	8.37	
No-tumor	1	18	374	2	94.68	5.32	90.78	9.22	
Pituitary Tumor	0	3	3	821	99.27	0.73	97.27	2.73	

**Table 3:** Performance of PSO-ReliefF KAZE-trained SVM model.

Class	Classified as				TPR (%)	FNR (%)	PPV (%)	FDR (%)	Accuracy (%)
	Glioma Tumor	Meningioma Tumor	No- Tumor	Pituitary Tumor					
Glioma Tumor	788	34	0	4	95.40	4.60	96.81	3.19	95.02
Meningioma Tumor	18	766	25	13	93.19	6.81	91.96	8.04	
No-tumor	8	24	357	6	90.38	9.62	92.97	7.03	
Pituitary Tumor	0	9	2	816	98.67	1.33	97.26	2.74	

**Table 4:** Performance of PSO-ReliefF SURF+KAZE trained SVM model.

Class	Classified as				TPR (%)	FNR (%)	PPV (%)	FDR (%)	Accuracy (%)
	Glioma Tumor	Meningioma Tumor	No- Tumor	Pituitary Tumor					
Glioma Tumor	792	33	0	1	95.88	4.12	98.02	1.98	96.30
Meningioma Tumor	14	775	20	13	94.28	5.72	93.94	6.06	
No-tumor	2	15	375	3	94.94	5.06	94.22	5.78	
Pituitary Tumor	0	2	3	822	99.40	0.60	97.97	2.03	

**Table 5:** Performance comparison of the proposed model with literature.

Study	Methodology	Accuracy (%)
Afshar et al. [50]	CNN	90.89
Cheng et al. [24]	Intensity histogram, gray level co-occurrence Matrix, and bag-of-words	91.28
Irmak. [16]	Deep learning model	92.66
Kang et al. [25]	Deep features	93.72
Almalki et al. [26]	SURF and KAZE	95.33
Alanazi et al. [19]	Pre-trained deep learning model	95.75
Rehman et al. [51]	Pre-trained deep learning model	95.86
Proposed Model	PSO-ReliefF SURF + KAZE	96.30

## Data Availability

Not applicable.
